# Factors affecting the microbiome of *Ixodes scapularis* and *Amblyomma americanum*

**DOI:** 10.1371/journal.pone.0232398

**Published:** 2020-05-15

**Authors:** R. Jory Brinkerhoff, Chris Clark, Kelly Ocasio, David T. Gauthier, Wayne L. Hynes

**Affiliations:** 1 Department of Biology, University of Richmond, Richmond, Virginia, United States of America; 2 School of Life Sciences, University of KwaZulu-Natal, Pietermaritzburg, South Africa; 3 Department of Biological Sciences, Old Dominion University, Norfolk, Virginia, United States of America; University of Minnesota, UNITED STATES

## Abstract

The microbial community composition of disease vectors can impact pathogen establishment and transmission as well as on vector behavior and fitness. While data on vector microbiota are accumulating quickly, determinants of the variation in disease vector microbial communities are incompletely understood. We explored the microbiome of two human-biting tick species abundant in eastern North America (*Amblyomma americanum* and *Ixodes scapularis*) to identify the relative contribution of tick species, tick life stage, tick sex, environmental context and vertical transmission to the richness, diversity, and species composition of the tick microbiome. We sampled 89 adult and nymphal *Ixodes scapularis* (N = 49) and *Amblyomma americanum* (N = 40) from two field sites and characterized the microbiome of each individual using the v3-v4 hypervariable region of the 16S rRNA gene. We identified significant variation in microbial community composition due to tick species and life stage with lesser impact of sampling site. Compared to unfed nymphs and males, the microbiome of engorged adult female *I*. *scapularis*, as well as the egg masses they produced, were low in bacterial richness and diversity and were dominated by *Rickettsia*, suggesting strong vertical transmission of this genus. Likewise, microbiota of *A*. *americanum* nymphs and males were more diverse than those of adult females. Among bacteria of public health importance, we detected several different *Rickettsia* sequence types, several of which were distinct from known species. *Borrelia* was relatively common in *I*. *scapularis* but did not show the same level of sequence variation as *Rickettsia*. Several bacterial genera were significantly over-represented in *Borrelia*-infected *I*. *scapularis*, suggesting a potential interaction of facilitative relationship between these taxa; no OTUs were under-represented in *Borrelia*-infected ticks. The systematic sampling we conducted for this study allowed us to partition the variation in tick microbial composition as a function of tick- and environmentally-related factors. Upon more complete understanding of the forces that shape the tick microbiome it will be possible to design targeted experimental studies to test the impacts of individual taxa and suites of microbes on vector-borne pathogen transmission and on vector biology.

## Introduction

Microbial communities associated with arthropod disease vectors have been a focus of study for over a decade (eg [1, 2, 3, 4, 5, 6]). Low-cost, high-throughput parallel sequencing has facilitated rapid growth in metagenomic studies (reviewed in [7]). While the majority of metagenomic studies of disease vectors focus on bacterial communities, rather than eukaryotic microbes or viruses, there is still much to learn about the factors that affect bacterial community composition in arthropod vectors and how these microbial communities might impact pathogen transmission [7]. In addition to the obvious importance of pathogen occurrence, abundance, and genotypic diversity in disease vectors, there is increasing recognition that non-pathogenic microbes might affect acquisition and transmission of vector-borne pathogens [8, 9]. For example, the presence of specific bacterial genera may inhibit the growth of pathogens, and some non-pathogenic bacteria may facilitate colonization by pathogens or affect vector behavior in ways that impact transmission dynamics [10, 11]. In other cases, pathogen occurrence may enhance vector survival [12, 13] and thus affect range expansion and abundance of such vectors in the environment [9, 14, 15].

The public health and veterinary importance of ticks makes this group of vectors highly attractive candidates for microbiome analysis. Ticks transmit a variety of bacterial pathogens to humans (*Borrelia* spp, *Francisella tularensis*, *Coxiella burnettii*) and livestock (*Ehrlichia* spp., *Anaplasma* spp.); they also serve as vectors for eukaryotic (*Babesia* spp., *Theileria* spp.) and viral (Crimean-Congo hemorrhagic fever virus, tick-borne encephalitis virus) pathogens. Although enzootic transmission for some of these pathogens is reasonably well-understood (eg tick-borne encephalitis, [16]), the roles of opportunistic or symbiotic bacteria in pathogen transmission are much less clear [17, 7]. Some intriguing data on the inhibitory or faciliatory roles of non-pathogen microbes in vector-borne disease dynamics is emerging. Ticks treated with antibiotics were less able to transmit *Borrelia burgdorferi* [10] and acquisition of *Anaplasma marginale* may be facilitated by the presence of *Rickettsia bellii* in *Dermacentor andersoni* [11]. Presence or absence of particular microbial species can affect vector survival: *Anaplasma phagocytophilum* may stimulate production of an antifreeze glycoprotein that improves freeze tolerance in the Lyme borreliosis vector, *Ixodes scapularis* [12]; this mechanism of increased pathogen survival may emerge by pathogen manipulation of the gut microbiome [18]. Similarly, *B*. *burgdorferi* (sensu lato) infection in *I*. *ricinus* affects host-seeking behavior [19], improves survival under stressful environmental conditions [13], and is associated with higher fat content in nymphal ticks [20]. Manipulation of the tick microbiome can also impact tick fitness, potentially opening opportunities for biocontrol applications [21, 22].

Drivers of tick microbiome composition are variable and likely to depend on tick species and study system. For example, variation in the tick microbiome has been noted in comparative studies of multiple species within (eg [23]) and among genera [24, 25]. Similarly, tick life stage is an important determinant of microbial community composition and bacterial species diversity in a variety of species [26], potentially due to different host utilization at different stages, or the accumulation of environmental bacteria over time. Sampling location may account for much of the microbiome variation, especially over large spatial scales (eg [27, 23]. Whether this variation is due, in part, to soil composition (eg [28]) or other environmental factors is not clear. In some studies, blood-feeding affected the relative abundance of different bacterial lineages, but not overall species diversity or richness [29]. In another study, blood feeding strongly shaped microbial community structure as well as richness and diversity [30], suggesting certain microbial genera react to the presence of blood by increasing in relative abundance. The host from which a bloodmeal originates also impacts microbiome composition [29, 30] just as host immune factors in blood can affect the persistence of certain pathogenic microbes [31, 32, 33]. However, different tick species feeding on the same host do not have identical microbiomes [25], suggesting that some of the tick microbiome is species-specific. Plant- and soil-associated bacteria are commonly detected in tick microbiota, contributing to site-specific heterogeneity in microbial community composition, but it is not clear whether these bacteria are part of the tick gut microbiome or whether they are contaminants from the body surface [34, 28]. Broadly, effects of host and environmental drivers of tick microbiome are not well understood.

Our goal for this study was to conduct targeted, stratified sampling to assess the relative impacts of tick species, sampling site, and life stage on microbiome composition in field-sampled *Amblyomma americanum* and *I*. *scapularis*, two common tick species in eastern North America that are of high public health concern [35, 36]. To assess potential vertical transmission of the microbiome, we conducted a second series of analyses on mated male and engorged female *I*. *scapularis* as well as on the egg masses produced by the engorged female ticks. Specifically, our first objective was to use a fully-factorial sampling design to partition the effects of tick species, sampling site, and life stage on microbial diversity and species richness in ticks. Our second objective was to characterize the microbiome of *I*. *scapularis* egg masses compared to engorged females and the males with which they mated to determine the relative contributions of male and female microbiota to the bacterial communities associated with egg masses.

## Materials and methods

### Sampling

**Fig 1:**
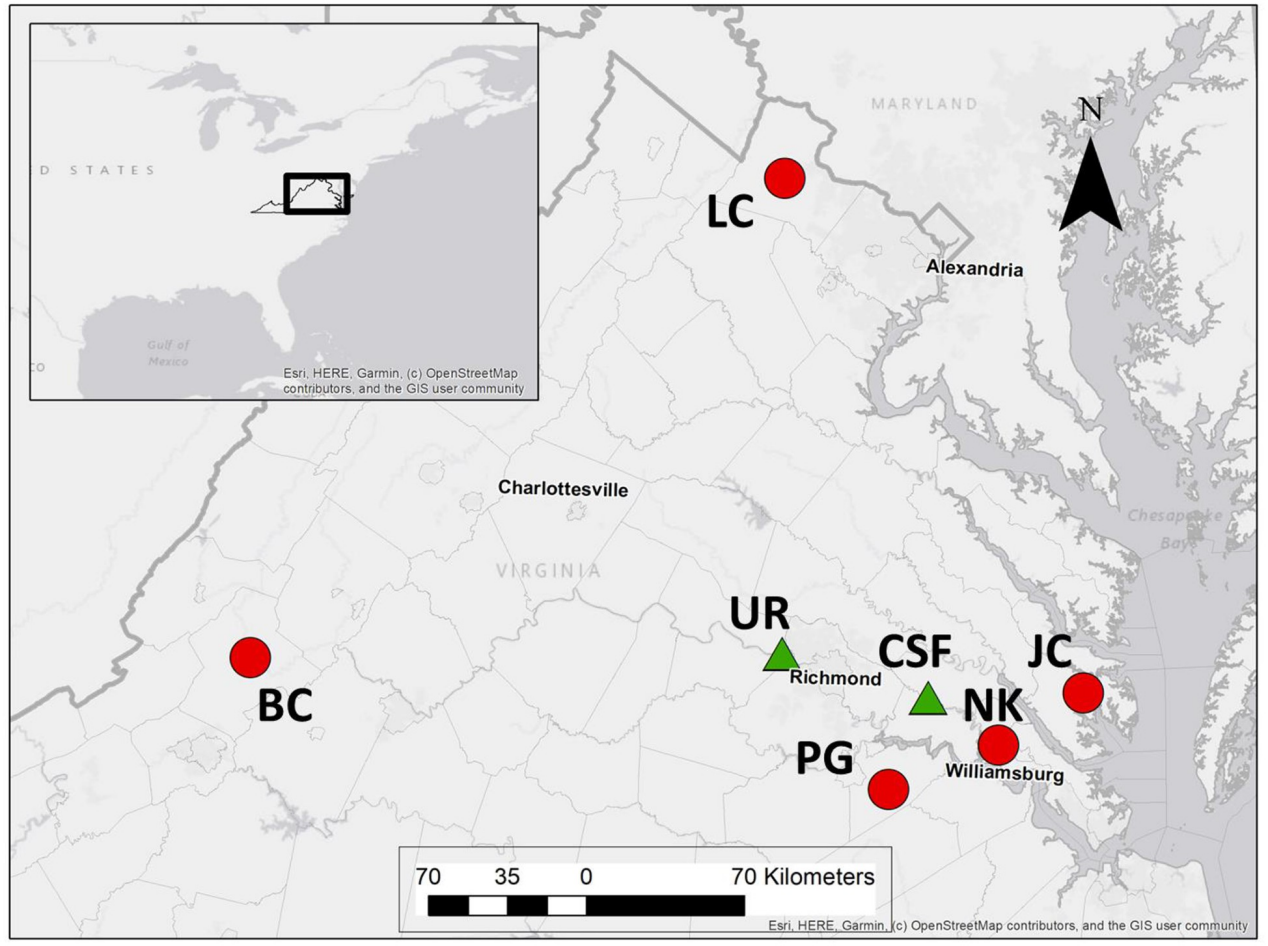
Location of study area and study sites in Virginia, USA. Red circles indicate sampling sites for host-derived adult *Ixodes scapularis* and green triangles represent locations from which host-seeking *I*. *scapularis* and *Amblyomma americanum* were collected. Site labels are as follows: BC = Botetourt County, LC = Loudon County, UR = University of Richmond property, PG = Prince George County, CSF = Crawfords State Forest, NK = New Kent County, JC = James City County. Site coordinates can be found in S1 Table.

Ticks were collected during two periods and using different methods to meet the objectives of this study. For analysis of host-seeking ticks, ticks were sampled from two field sites in east-central Virginia by drag and flag sampling in May and June 2017 (Fig 1). Sampling sites were selected based on occurrence of established populations of both *Amblyomma americanum* and *Ixodes scapularis* with each site existing in a different physiographic region: coastal plain and piedmont plateau. Previous analyses have shown that *B*. *burgdorferi* prevalence in *I*. *scapularis* nymphs varies between these regions and *I*. *scapularis* population genetic histories differ significantly between the coastal plain and populations farther to the west [37, 38], possibly indicating differences in the underlying metagenomic profiles of ticks at these sites. Microbiota of individual ticks from the same population may vary substantially [39] so our goal was to collect ten adult male, ten adult female, and ten nymphal *Ixodes scapularis* and the same number of *Amblyomma americanum* from each site and we visited each site on several occasions to maximize our chances of meeting this objective. Collected ticks were placed in 70% ethanol and stored at -20 degrees C prior to DNA extraction.

To assess consistency of the microbiome from engorged females to the egg masses they produce, we collected engorged females and, when possible, mated males, from white-tailed deer (*Odocoileus virginianus*) carcasses processed at Burks Farm in New Kent County, Virginia. Carcasses for which locality data were available were searched for engorged female *I*. *scapularis* ticks with males attached. Pairs of ticks were removed with fine-pointed forceps and placed in individual sterile 10 ml glass tubes sealed with mesh fabric. Live ticks were held in a desiccator at 21 degrees C and 90–95% humidity until egg masses were produced. After oviposition, egg masses were separated from adult ticks using sterile 25 gauge needles and all samples were frozen in separate tubes at -80 degrees C. Prior to DNA extraction, all ticks (both species, all life stages except eggs) were washed twice with 95% ethanol. Egg masses were not washed with ethanol so as to preserve as much sample material as possible. No permits were required for collection of the samples in this study and permission to collect samples was granted by the owner-operators of the deer processing facility from which deer-derived ticks were collected and from the Virginia Department of Forestry (drag-sampled ticks).

### DNA extraction, library preparation, and sequencing

Ticks were flash frozen in liquid nitrogen and pulverized in individual microcentrifuge tubes using sterile pestles; egg masses were not pulverized. DNA was extracted from individual ticks and egg masses using a Nucleospin Soil kit (Macherey-Nagel, Inc., Bethlehem, PA, USA) following the manufacturer’s protocol. Bacterial 16S rDNA was amplified from each sample by targeting the v3-v4 hypervariable regions of the 16S gene following the protocol outlined in the 16S Metagenomic Sequencing Library Preparation manual [40]. Briefly, following the initial 16S PCR, amplicons were purified using AMPure XP beads (Beckman Coulter Life Sciences, Indiana, USA). Barcodes were added to individual samples using Nextera XT Index kit using PCR conditions in the 16S library preparation manual [40]. Resulting amplicons were again purified using AMPure XP beads and the amplicon size was measured using an Agilent Bioanalyzer (Agilent Technologies, Inc., Santa Clara, CA, USA) using the high-sensitivity DNA protocol. DNA concentrations were determined using a Qubit spectrophotometer and normalized to ensure equal representation among samples, and then pooled to a final concentration of 4 nM. Sequencing was conducted on an Illumina MiSeq using a MiSeq V3 2 x 300 bp reagent kit (Illumina, Inc., California, USA). Sequences reported here were generated from two separate MiSeq runs, each identical in library preparation and sequencing protocol.

### Informatic and statistical analysis

Raw fastq files were first paired by sample/index with forward and reverse reads merged into individual contigs, grouped by sample/index. Paired reads were screened for quality and filtered by read length to an average of 446 basepairs, reduced to 440 basepairs after quality trimming. Operational taxonomic units (OTUs) were characterized using QIIME [41, 42]; OTUs were assigned using open-reference picking and taxa were assigned from the Greengenes 8_13 reference database. Sequences that showed similarity of at least 97% were then clustered into the same OTU. We also used QIIME to analyze alpha and beta diversity. We used adonis2 in the R [43] package vegan 2.4.2 [44] to perform permuted multiple analyses of variance (perMANOVA) to test for variation in microbiome composition by site, tick genus, and life stage, and sex as well as to compare microbial taxa composition between engorged females, egg masses, and mated males. For the purposes of these analyses, we define life stage as egg mass, nymph, adult male, and adult female; male and female are not different life stages, but because sex cannot be differentiated at the nymph or egg mass stage, we found it convenient to include sex information within the adult life stage rather than include a separate variable encoding sex. Where possible, we used *post hoc* testing to make comparisons of microbial community composition among life stages (as defined here).

In addition to using raw OTU richness counts for each sample, we rarefied OTU counts using the *rarefy* command in R package vegan [44] to account for variable sequencing depth. We calculated Shannon’s diversity index for each sample and analyzed variation in bacterial diversity and richness with linear models in R. We performed indicator species analysis using PC-Ord 7 [45] to test for non-random associations between *Borrelia* and other bacterial taxa in *Ixodes* samples. Briefly, samples were coded as *Borrelia* positive or negative and the relative abundance of each other taxon in samples belonging to each group is multiplied by the relative frequency of that taxon in each sample in that group to generate an indicator value (IV). Monte Carlo randomizations (N = 15,999) were then run to generate distributions of randomized IVs for each taxon in each group; statistical significance is determined based on comparisons between observed and expected IVs under the null hypothesis of no associations between taxa and group assignment. We adjusted the critical p-value to account for analysis of each of 492 OTUs in this dataset. We explored the overlap in core OTUS, defined initially as OTUs that were present in at least 80% of samples of a given group and at 1% minimum relative abundance [46], between engorged females, egg masses, and mated males. To explore the impact of *Rickettsia* on microbiome diversity estimates, we removed all reads assigned to *Rickettsia*, re-calculated diversity metrics, and re-ran diversity analyses. Finally, we assessed sequence variation and diversity of selected bacterial lineages (*Rickettsia* and *Borrelia*) using MEGA 7 [47] by first aligning OTU sequences from our dataset with reference sequences downloaded from GenBank and calculating the percent match of each OTU we detected to the reference sequences.

## Results

We collected 27 adult *A*. *americanum* (13 female and 14 male), 21 *A*. *americanum* nymphs, 3 adult *I*. *scapularis* (two female, one male), and 18 *I*. *scapularis* nymphs by drag sampling (S1 Table). Consistent with previous sampling efforts [37, 38], we had difficulty collecting adult *I*. *scapularis* is eastern Virginia during spring and summer months; as a result, we did not have equal representation of all life stages from each sampling site for *I*. *scapularis*. Thirteen engorged female *I*. *scapularis* collected from deer produced egg masses. Of these females, eight were mated to males at the time of collection. Egg masses were laid within 5 weeks of tick collection and in one case, an egg mass hatched into larvae prior to being frozen. PCR and library preparation was carried out on 92 samples (S1 Table). Microbiome data were obtained from 90 samples, 89 of which provided more than 1000 sequences (range = 1,051–932,918, mean = 67,703, median = 32,851; Table 1 and S1 Table); for subsequent analyses, we discarded sample CC51F which produced only 230 reads. For rarefied estimates of OTU counts we used a sample of 1,051 sequences per tick, corresponding to the cutoff value described above. We detected a total of 3,672 operational taxonomic units (OTUs), or unique sequences, in our dataset prior to clustering. When classified at the genus level, using the GreenGenes database where sequences are clustered at 97% similarity, we were left with 546 OTUs. Sequences that could not be characterized as any taxon within the Kingdom Bacteria (N = 41,010) were grouped into a single “unassigned” category. Except where noted, all analyses were conducted on the genus-level dataset of 546 OTUs.

**Table 1:** Sample size, average sequencing depth, OTU richness and OTU diversity for each life stage and each tick species.

Species	Stage	N	Sequences (mean)	Richness (mean)	Diversity (mean)
*A*. *americanum*					
	Female	11	102,392	86.0	1.66
	Male	9	15,097	99.1	2.64
	Nymph	20	62,906	116.9	2.34
	*TOTAL*	*40*	*63*,*008*	*104*.*4*	*2*.*22*
*I*. *scapularis*					
	Egg mass	12	107,993	46.5	0.71
	Female (fed)	12	98,857	39.6	0.55
	Female (unfed)	2	15,616	131.0	3.39
	Larval mass	1	35,752	48.0	0.66
	Male	7	67,392	88.6	2.45
	Nymph	16	34,500	111.3	2.70
	*TOTAL*	*50*	*71*,*459*	*74*.*9*	*1*.*66*
**Grand Total**		**90**	**67,703**	**88.0**	**1.91**

### Microbial community structure

**Fig 2:**
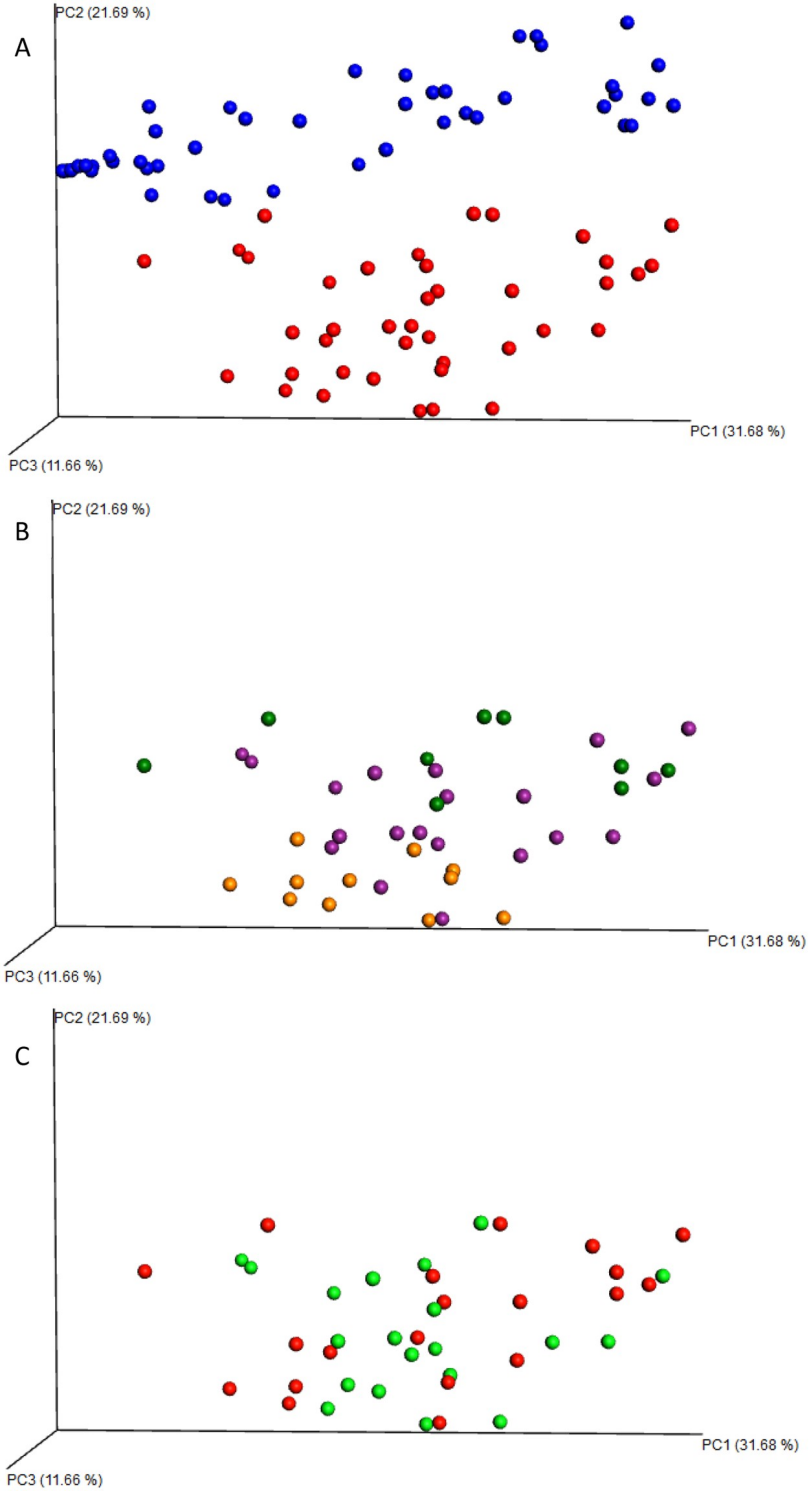
Weighted unifrac plots differentiating samples by A) tick species, B) life stage site (only *A*. *americanum* data shown; males in green, nymphs in purple, and females in orange), and C) sampling site (only *A*. *americanum* data shown; red represents site CSF and green represents site UR). The entire dataset is found in S1 Dataset and can be downloaded and manipulated to highlight subsets of data related to any variable in the metadata file.

Microbiome composition varied significantly by tick species (PerMANOVA: F = 24.68, P = 0.0001; Fig 2A), life stage (PerMANOVA: F = 4.43, P = 0.001; Fig 2B), but not by sampling site (PerMANOVA: F = 2.23, P = 0.051; Fig 2C); there was also a significant site-by-tick species interaction (PerMANOVA: F = 2.93, P = 0.014). Because we did not have equal representation of all life stages of each species at each site, we performed a restricted analysis using only host-seeking nymph samples and found that species (PerMANOVA: F = 14.38, P = 0.0001) and sampling site (PerMANOVA: F = 2.51, P = 0.03) as well as the interaction between site and species (PerMANOVA: F = 2.53, P = 0.027) were all important determinants of OTU composition. For *A*. *americanum* only, where we had sufficient representation of each life stage and both sexes at both life sites, stage (PerMANOVA: F = 5.26, P = 0.0001) and site (PerMANOVA: F = 3.40, P = 0.011) were significant predictors of the tick microbiome but the interaction between site and stage was not (PerMANOVA: F = 1.39, P = 0.20). When comparing microbial composition among *I*. *scapularis* life stages (engorged female, mated male, nymphs and egg mass), we found significant variation by life stage (PerMANOVA: F = 5.03, P = 0.004) as well as significant variation in OTU richness (PerMANOVA: F = 19.32, P≪0.0001, r2 = 0.55) and diversity (PerMANOVA: F = 37.38, P≪0.0001, r2 = 0.71) among life stages.

### Microbial richness and diversity

Rarefied OTU richness varied significantly by life stage for *Ixodes* (F = 19.95, P<0.001, adjusted R2 = 0.55) with nymphs (T = 6.81, P<0.0001) and males (T = 3.91, P = 0.008) having greater richness than females and eggs. For *Amblyomma*, where we could model OTU richness as a function of site and life stage, there was no effect of either predictor, or the interaction term, on the rarefied OTU richness (F = 2.28, P = 0.068, adjusted R2 = 0.014). OTU diversity in *Ixodes* varied significantly by life stage (F = 18.11, P<0.0001, R2 = 0.55), with nymphs (t = 6.19, P<0.001) and males (t = 4.35, P<0.001) having highest OTU diversity. OTU diversity in *Amblyomma* also varied significantly as a function of site and stage (F = 2.86, P = 0.029, adjusted R2 = 0.19) where males (T = 2.30, P = 0.028) and nymphs (T = 2.95, P = 0.006) had higher OTU diversity than females. In a nymphs-only analysis, there was no effect of site, species, or the interaction between the variables on either rarefied OTU richness (F = 1.65, P = 0.20, adjusted R2 = 0.05) or diversity (F = 2.83, P = 0.054, adjusted R2 = 0.14). When all host-seeking ticks (ie exclusive of *Ixodes* collected from deer) were included in a single analysis, rarefied OTU richness did not depend on tick species, sampling site, or life stage (F = 1.65, P = 0.18, adjusted R2 = 0.04). In all analyses, results from tests of raw OTU richness counts were qualitatively similar to those of rarefied OTU estimates.

**Fig 3:**
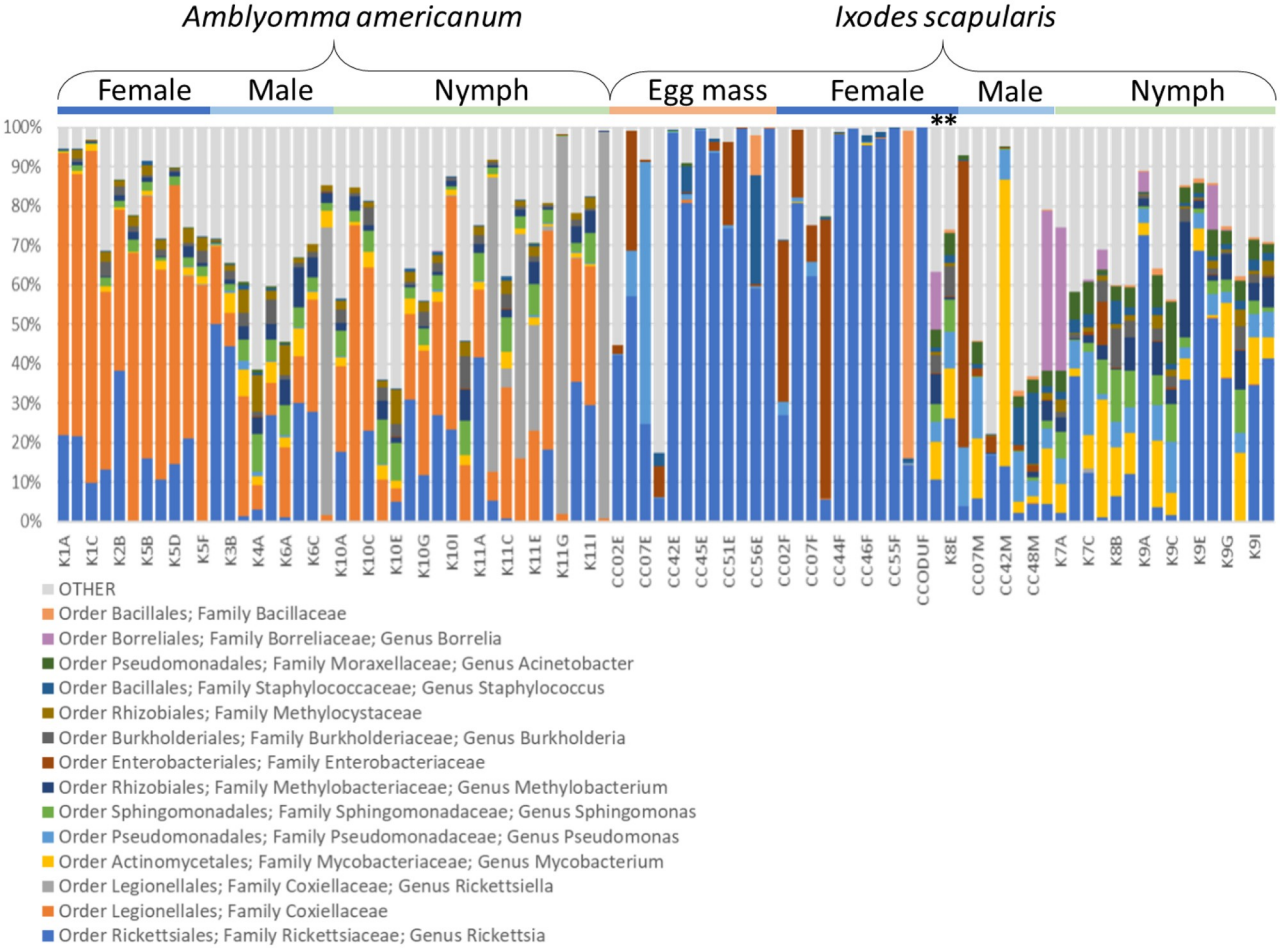
Relative abundance of the 15 most commonly-detected OTUs in this study, organized by tick species and life stage. Taxa (order, family, and genus where available) are listed below the figure. Sample numbers are listed alphabetically within a life stage group and areindicated along the x-axis. Specific samples can be identified either by their labels along the x-axis or, if unlabeled, can be derived from the list of samples (S1 Table). Asterisks indicate two unengorged host-seeking female ticks that were collected during drag sampling; all other female ticks included in this figure were engorged and the time of processing.

**Fig 4:**
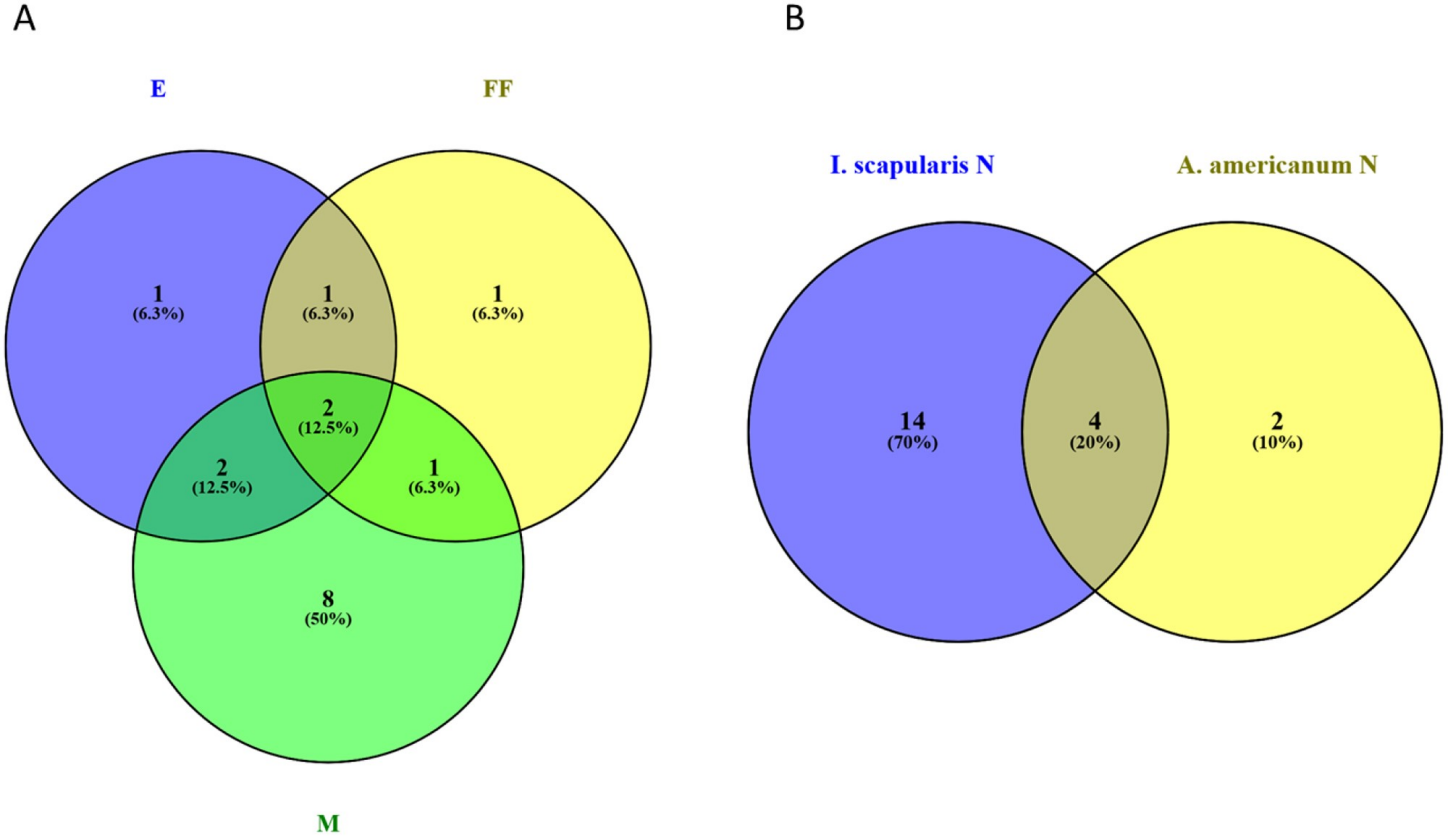
Core OTUs, defined as taxa present in at least 50% of samples within a group and occurring at 1% minimum relative abundance. Overlap A) among *I*. *scapularis* engorged females (FF), mated males (M), and egg masses (E) generated from deer carcasses and B) overlap between *Amblyomma* and *Ixodes* nymphs. Graphs were generated with Venny 2.1 (https://bioinfogp.cnb.csic.es/tools/venny/).

Qualitatively, it is apparent that OTU composition varies by tick species and life stage (Figs 2A, 2B and 3). For example, Coxiellaceae are the most common and abundant lineage in *A*. *americanum* whereas *Rickettsia* dominate engorged female *I*. *scapularis* and their egg masses (Fig 3). OTU variation and diversity (Table 1) in host-seeking female *I*. *scapularis* are similar to that of host-seeking males and nymphs. We detected very few OTUs that qualified as core OTUs under the initial definition (1 in engorged females, 4 in egg masses, and 8 in males, with one in common among all stages) so we broadened the definition to OTUs that occurred in at least 50% of samples and 1% minimum relative abundance and found that the core microbiota of eggs were largely similar to male and engorged female *I*. *scapularis* (Fig 4A). Although richness and diversity were equivalent among *A*. *americanum* and *I*. *scapularis* nymphs, there were 14 OTUs unique to *I*. *scapularis* nymphs and 2 unique to *A*. *americanum* nymphs, with 4 OTUs present in nymphs of both tick species (Fig 4B).

### Removal of *Rickettsia*

**Fig 5:**
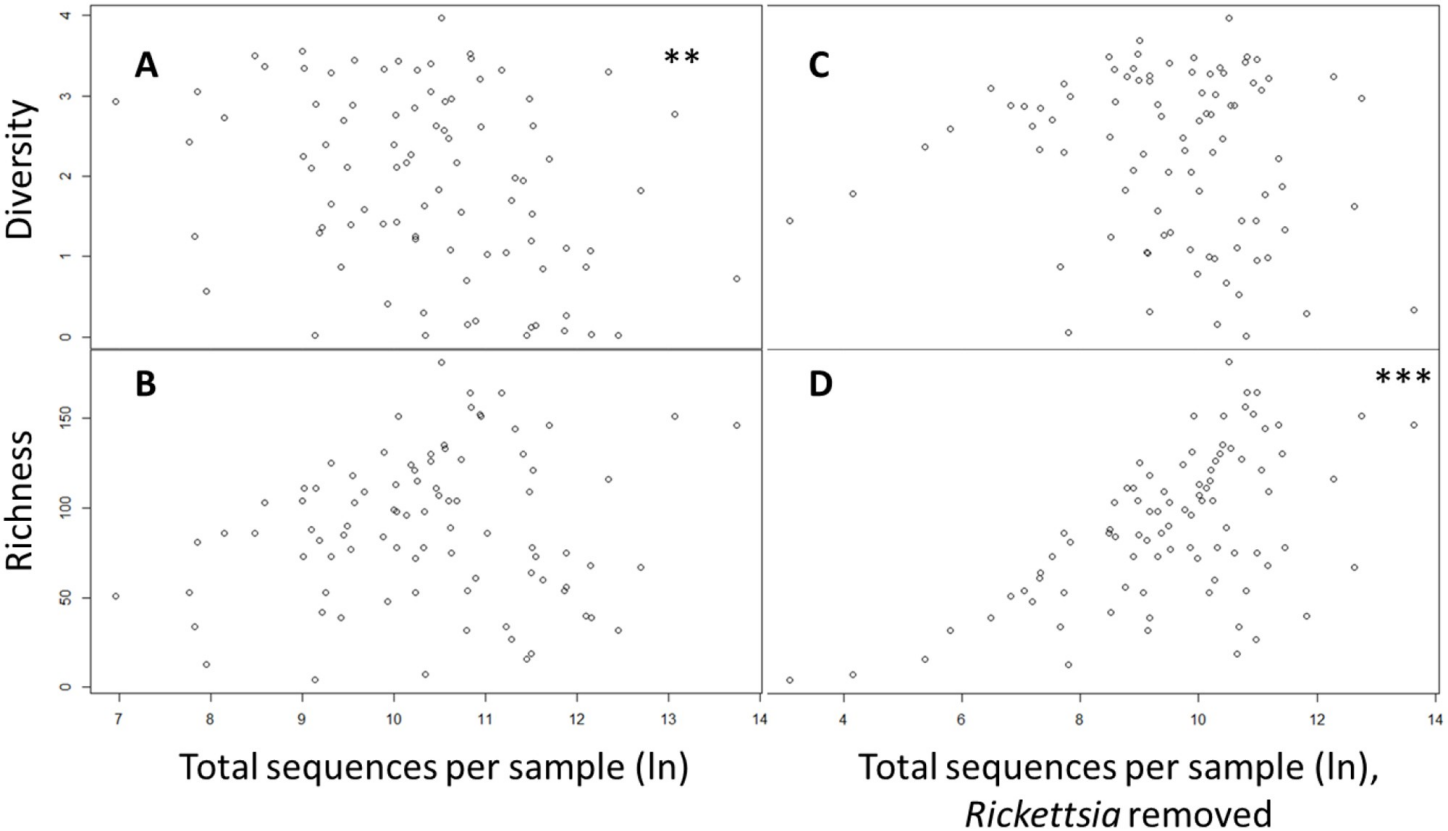
Relationship between sequencing depth per sample and microbial diversity (Shannon’s Index) in ticks with (A) and without (C) *Rickettsia* included in the analysis. Panels B and D show the relationship between sequencing depth and taxonomic richness with and without *Rickettsia*, respectively. Asterisks indicate significance at P = 0.01 (**) and P = 0.001 (***).

Because *Rickettsia* was present in every sample and dominated the microbiome of engorged female *I*. *scapularis* and *I*. *scapularis* egg masses (Fig 3), we re-ran all analyses after informatically removing *Rickettsia* sequences from the dataset. Comparisons of microbiome composition in host-seeking ticks were not qualitatively affected by this modification; we still found significant variation among species, life stage, and site, as well as genus and site-dependent variation when exploring the nymphs-only dataset. However, after removing *Rickettsia*, the microbiome composition of *I*. *scapularis* egg masses, engorged females, and males could no longer be differentiated (F = 0.822, P = 0.71), although OTU richness still varied significantly by life stage (overall F = 5.9, P = 0.003). Prior to removal of *Rickettsia*, linear regression analysis showed a weak but significant negative relationship between OTU diversity and the natural log of the number of sequences per sample (F = 8.87, P = 0.004, adjusted R2 = 0.08; Fig 5A). However, there was no relationship between the natural log of the number of sequences produced per sample and OTU richness (F = 1.78, P = 0.19, adjusted R2 = 0.01; Fig 5B). After removing *Rickettsia*, there was no dependence of OTU diversity on sequencing depth (F = 1.06, P = 0.31, adjusted R2 = 0.001; Fig 5C) but there was a strong and significant relationship between sequencing depth per sample and OTU richness (F = 42.8, P < 0.001, adjusted R2 = 0.32; Fig 5D). We note that the minimum number of reads per sample after removal of *Rickettsia* dropped to 22, so these results should be interpreted with caution. Rarefied OTU richness (F = 6.51, P = 0.014, adjusted R2 = 0.11) and OTU diversity (F = 4.96, P = 0.031, adjusted R2 = 0.08) were significantly higher in *I*. *scapularis* (all life stages included) that carried *Borrelia*. To test whether this was an effect of reduced OTU diversity in engorged females, where *Borrelia* prevalence and relative abundance is lower because of bacteriolytic effects of deer blood on *Borrelia*, we re-ran these analyses using only host-seeking *I*. *scapularis* nymphs (N = 16). For the nymphs-only analysis, neither rarefied OTU richness (F = 3.43, P = 0.09, R2 = 0.14) or diversity (F = 1.44, P = 0.25, adjusted R2 = 0.03) were dependent on *Borrelia* infection status (F = 6.51). We found significant over-representation of several genera in *I*. *scapularis* that were carrying *Borrelia* (Table 2).

**Table 2:** Taxa over-represented in *Borrelia*-positive ticks as identified by indicator-species analysis with 15,999 simulations, arranged by probability of null association. Indicator values (IV) represent the combined relative abundance of a given taxon in a group (*Borrelia* positive/negative) and the proportion of samples within that group that had that taxon; an IV of 100 indicates perfect association of a taxon with a group due to exclusive occurrence and high relative abundance. Significance is determined by comparison of observed IVs with IVs generated from randomizations. No taxa were identified as being under-represented in *Borrelia*-positive ticks. Results were qualitatively similar whether or not *Rickettsia* sequences were included in the dataset. Asterisks indicate statistical significance at P = 0.0001 with the two taxa associated with P ≤ 0.0005 listed as marginally-significant indicator taxa.

Taxon	Indicator Value (IV)	Probability of result under the null hypothesis of no association
*Borrelia*‡	100	0.0001*
*Tepidomonas*	77.7	0.0001*
*Luteibacter*	85.7	0.0001*
*Francisella*	58.3	0.0004
*Fibriimonas*	72.3	0.0005

^‡^This taxon was used to define the groups so an IV of 100 is an artefact of the analysis

We explored phylogenetic diversity of *Rickettsia* 16S DNA in our samples compared to other tick-associated and pathogenic *Rickettsia* species [48]. Twelve sequences were identified as genus *Rickettsia* but for phylogenetic analysis we used only the six sequences that were detected at least 200 times and in multiple ticks to reduce impacts of PCR or sequencing error. In addition to sequences similar to *Ixodes*- and *Amblyomma*-associated *Rickettsia* species from eastern North America (*R*. *buchneri*-like and *R*. *amblyommatis*-like, respectively), we detected several *Rickettsia* sequences that have sequences distinct from known lineages (Table 3). Similarly, we performed phylogenetic analysis of the two *Borrelia* sequences from our dataset compared to other known *Borrelia* species, however, a number of *B*. *burgdorferi* (eg N40, CA4, MM1, JD1) and *B*. *bisettii* (eg 13–80, DN127) strains are identical at this 440 bp portion of 16S rDNA.

**Table 3:** Percent identity of *Rickettsia* OTUs detected in this study to tick-associated *Rickettsia* and *Rickettsia* representing the three major *Rickettsia* clades: Spotted fever group (SFG), transitional group (TG), and typhus group (TRG) [4,8]. Each OTU included in this table is represented by at least 200 reads. Asterisks denote OTUs that were detected only in *I*. *scapularis*; all other OTUs were detected in both tick species.

	*A*. *americanum* and *I*. *scapularis-*associated *Rickettsia*				SFG		TRG	TG
OTU	*R*. *monacensis* LN794217.1	*R buchneri* NR_134842.2	*Rickettsia* sp. (*I*. *scapularis* symbiont) D84558.1	*R*. *amblyommatis* CP015012.1	*R*. *rickettsii* NR_118678.1	*R*. *parkeri* L36673	*R*. *felis* DQ102712.1	*R*. *typhi* NR_074394.1
2	100	100	100	99.48	99.48	99.48	99.48	99.48
874*	100	100	100	99.47	99.47	99.47	99.47	99.47
3009	99.2	99.2	99.2	98.67	98.67	98.67	98.67	98.67
1020*	99.45	99.45	99.45	98.9	98.9	98.9	98.9	98.9
1303	97.37	97.37	97.37	97.89	97.63	97.37	97.37	97.37
732	97.11	97.11	97.11	96.59	97.11	97.11	96.85	96.85

## Discussion

There is considerable interest in characterizing the microbiota of disease vectors [7], in part because interactions among bacteria may affect pathogen viability and transmission [10]. Tick microbiota are highly variable, particularly in the relative abundance of specific bacterial genera [26, 49, 29, 30]. Previously identified factors associated with variation in tick microbiome composition include: tick species [23], sampling location and environmental conditions [23, 28], engorgement status and host bloodmeal [30], tick life stage [27], and tick age [50]. However, the effects of these factors are not always consistent. For example, host species identity affected *I*. *pacificus* microbiome in one study [30] but did not significantly affect tick microbiome in another [24]. Some of this heterogenetiy among studies could result from analysis of different molecular targets [51], stochastic variation among individual ticks [39], or methodological approaches regarding sample preparation or sequence processing and analysis. Our goal for this study was to compare the relative importance of environmental and organismal predictors of the microbiome composition of two common human-biting tick species in eastern North America using a stratified and factorial study design.

### Effects of species and life stage on the tick microbiome

Tick species identity accounted for the largest portion of microbiome variation in our study, although life stage was also a major determinant of bacterial species composition for each tick. This is consistent with previous studies [52] where the microbiota of lab-reared and field-sampled *Ixodes scapularis*, fed on different host species, were compared and shown to vary as a function of environment more than host species identity. Similarly, Rynkiewicz et al. [25] described significant microbiome variation between *Ixodes scapularis* and *Dermacentor variabilis* larvae, even when ticks fed on the same individual hosts. Immature *I*. *scapularis* and *A*. *americanum* do not generally overlap in host species use, but as adults, both species commonly parasitize white-tailed deer (*Odocoileus virginianus*) [53]. If host species identity was a strong determinant of tick microbiome, we might expect convergence of microbial community composition when comparing engorged *I*. *scapularis* to *A*. *americanum*, but this pattern was not observed (Fig 3). While the composition of the microbial community in our study varied significantly between *A*. *americanum* and *I*. *scapularis* (Figs 2A and 3), OTU richness and diversity did not when we restricted our analyses to the same life stage (nymphs). Within species, however, richness and diversity did tend to vary by stage and by sex with male and nymphal ticks having higher richness and diversity than female ticks, whether engorged or host-seeking, though we note the small number of host-seeking adult *I*. *scapularis* collected during dragging (Table 1). Interestingly, this pattern was observed in *A*. *americanum* where females were unfed, suggesting the lower diversity and richness of OTUs is not a simple artefact of bloodfeeding. A previous study of the *A*. *americanum* microbiome [26] also reported lower microbial diversity in females than males or nymphs, and a similarly high relative abundance of *Rickettsia*, particularly in females. Williams-Newkirk et al. [49] also found the microbiome in *A*. *americanum* to vary by life stage and by sex for adult ticks with the microbiome of all stages dominated by *Rickettsia* and *Coxiella*, as in our study. In addition to life stage, tick age may affect microbial diversity and richness in *Amblyomma americanum* with the microbiome apparently losing taxa from time of molting from nymph to adult to 100 days post-molt [50]. For *I*. *scapularis*, eggs and females had similar microbial richness, diversity, and species composition, suggesting potential vertical transmission of microbes from a female to her offspring. We do not know for certain whether the microbiota detected in egg masses were on or inside of the eggs themselves, and cannot conclude that these bacteria were definitively vertically transmitted. However, one larval mass (CC46L; S1 Table) was included in our analysis and it was also dominated by *Rickettsia*, clustering tightly with egg masses and engorged females in unifrac analysis (S1 Dataset). Moreover, Zolnik et al. [52] demonstrated that larvae emerged from egg masses with high relative abundance of *Rickettsia*, which is also consistent with vertical transmission of this genus. Of the six core OTUs detected in egg masses, five were also core OTUs for males (N = 2), engorged females (N = 1), or common to both adult sexes (N = 2) with only one unique core OTU (Fig 4A). Similarly, engorged females were found to have only one unique core OTU whereas males had eight. Whether the overlap in core OTUs between egg masses and the adults from which they were generated and fertilized indicates vertical transmission is to be determined, but the relative paucity of characteristic core OTUs in engorged females and eggs is noteworthy.

### Effects of local environment on the tick microbiome

There was substantial variation in microbiome composition within a site and, on average, sampling location explained very little of the variation in microbial community structure and did not affect bacterial richness or diversity. We do not have data on host bloodmeal identification for the ticks we tested, with the exception of the adult *I*. *scapularis* females removed from deer, and it is possible that different host use accounts for some of the spatial heterogenetiy in microbiome composition we detected. Controlled experiments whereby microbiome varied among lab-reared immature ticks were fed on various hosts supports this speculation (eg [25, 30]. Other studies have demonstrated that lab-reared ticks have depauperate and distinct microbiomes when compared to field-collected individuals of the same species [52, 54], suggesting that existence in the natural environment drives accumulation of specific microbial taxa, whether or not colonization occurs through feeding.

It is possible that the overall similarity in tick microbiota between sites in our study was due to the relative proximity (< 100 km) of our sites to each other. Variation between sampling sites at larger spatial scales have shown strong geographical differences in the microbiome composition of particular tick species [23, 39]. Trout-Fryxell and DeBruyn [28] found significant differences in the microbiota of *A*. *americanum* among sites that had different soil types and drainage, suggesting local environmental conditions that affect soil and environmental bacteria (eg [55]) can also affect tick microbiota. Given that much of the tick microbiome is likely environmentally-derived rather than host-derived [52, 30, 54], it is reasonable to assume that regional variation in environmental bacteria would lead to differences in local tick microbiota. We expected some variation in tick microbiota between the coastal plain and piedmont plateau, but the within-site variation in microbiota masked any potential differences in microbial community composition between sites. Our results indicate that relatively little of the core microbiome is shared between species (Fig 4b) when life stage is held constant, suggesting there are bacteria specifically adapted to different tick genera or species.

As with any community sampling approach, there is a trade-off between accurate representation of species occurrence and relative abundance and sampling effort. Rarefaction can be used to mitigate some of the effects of unequal sampling effort (here, sequencing depth) among samples; our results were equivalent with rarefied and non-rarefied data. However, it is important to acknowledge the effects of sequencing depth on measurement of species richness and diversity. When all samples were analyzed together, there was no effect of sequencing depth on detected OTU richness but there was a weak negative relationship between sequencing depth and OTU diversity. This suggests we characterized the microbiome of our ticks reasonably well and that increased sequencing depth tends to lead to the accumulation of sequences from the most abundant OTUs. However, when we removed the most abundant OTU (OTU2, *Rickettsia*) from our dataset there was a significant positive relationship between sequence depth and OTU richness, particularly at lower sequencing depth (Fig 5d). Thus, for samples dominated by *Rickettsia* and for which sequencing depth was low, we likely failed to detect less abundant OTUs. This information is useful for design of future studies. With *a priori* knowledge or suspicion of low-diversity samples due to dominance by one or two OTUs, added sequencing depth, achievable by enrichment of such samples in pooled sequencing libraries, may result in better characterization of the composition of the remaining microbiome, especially after informatic removal of the most abundant taxa.

### *Rickettsia* and *Borrelia*

In our study, the microbiota of blood-fed female *I*. *scapularis* were dominated by *Rickettsia*, as were their egg masses. This result is consistent with vertical transmission of this taxon, as has been shown in previous studies [52, 56]. The observation that engorged females had lower microbial richness than both males and nymphal ticks suggests that blood-feeding may allow for increased populations of *Rickettsia*, potentially at the expense of maintaining other lineages. Although we tested relatively few unengorged females for comparison to engorged females, both clustered with male and nymphal *I*. *scapularis* in terms of microbiome composition (S1 Dataset). Given that male and nymphal *I*. *scapularis* showed similar microbial richness and diversity, and that the two unengorged female *I*. *scapularis* showed microbial profiles similar to the other unengorged stages (Fig 3), we suggest that bloodfeeding has a strong impact on microbial richness and diversity. The relative lack of unique taxa in engorged females when compared to males (Fig 4A) suggests that the remaining non-*Rickettsia* OTUs in these ticks represent a subset of the taxa found in other life stages. We speculate that blood-feeding serves to reduce microbial richness in *Ixodes* ticks, as has been demonstrated previously [30], with the ticks subsequently being colonized by environmental bacteria that then restore the microbiome to levels of richness detected in host-seeking ticks. However, we cannot rule out the possibility that the disappearance of some microbial genera in engorged female microbiota is artefactual. The low relative abundance of certain OTUs may preclude their detection after increases in the populations of other bacteria (eg *Rickettsia*) during and after blood-feeding. Several engorged females yielded relatively high sequence depth (ie >100,000 sequences; S1 Table) and still produced relatively little OTU richness, suggesting true absence rather than failure to detect certain bacterial genera.

*A*. *americanum* is associated with a number of *Rickettsia* species, both pathogenic and purportedly endosymbiotic [57, 58, 59, 60]. *Rickettsia parkeri*, the agent of tidewater spotted fever, has been detected in *I*. *scapularis* [61], but we are aware of no data indicating its ability to transmit this agent. Far more common in *I*. *scapularis* are *Rickettsia* similar or identical to *R*. *buchneri*, [62, 63, 64], a species whose pathogenic capacity is undescribed. We were are unable to confidently identify the *Rickettsia* in our study to species because of the low phylogenetic resolution associated with the portion of the v3-v4 region of 16S rRNA gene we analyzed. Moreover, *R*. *monacensis*, a species indistinguishable from *R*. *buchneri* and other species based on the locus used in this study, can be acquired through feeding by *A*. *americanum*, though it does not disseminate from the gut the way it does in *I*. *scapularis* [65]. Detection of bacterial DNA in a tick does not indicate bacterial viability or ability of the tick to transmit, and we stress that we are not able to definitively identify these *Rickettsia* to species; the most commonly-detected OTU in our study (OTU2, Table 3) is identical to several tick-associated *Rickettsia* including *R*. *buchneri* and *monacensis*, as well as a purported symbiont of *I*. *pacificus* (Table 3). Interestingly, we detected this OTU in both tick species and we did not find any OTUs that matched exactly to *R*. *amblyommatis* (*amblyommii*) type strains Ac37 or An13. However, these type strains were isolated from different *Amblyomma* species (*A*. *cajennense* and *A*. *neumanni*, respectively), both in South America, and they only differed at two positions from OTU2 in our dataset. Irrespective of the species represented by OTU2, we did find that the genetic diversity of *Rickettsia* in our sample is relatively high with several of the sequences we detected differing from known from major *Rickettsia* groups (Table 3). Although *Rickettsia* are common endosymbionts of ticks and previous studies have demonstrated substantial genotypic diversity of *Rickettsia*-like organisms [66], our data suggest that there may be additional diversity of undescribed *Rickettsia* or *Rickettsia*-like organisms in both *A*. *americanum* and *I*. *scapularis* (Table 3). Further analyses of *Rickettsia* to the species level will require follow-up molecular analyses with makers that allow for higher-resolution phylogenetic analysis.

As was the case with *Rickettsia*, the segment of the 16S rRNA gene we used for this metagenomic analysis does not differentiate between *Borrelia* species; *B*. *burgdorferi* strain B31 and *B*. *bissettii* strain DN137 (among others) are identical at this locus and both species may be present at our study sites [67, 68, 37]. Prior to clustering, we identified two sequences that were classified as genus *Borrelia*: one (N = 1,579) matched exactly to the B31 type strain whereas the other (N = 18,628) had one polymorphism out of 440 nucleotide positions. In a previous metagenomic analysis of Virginia *I*. *scapularis*, all of the *Borrelia* sequences were confirmed to be *B*. *burgdorferi* upon sequence analysis of other loci in the *B*. *burgdorferi* genome [23]. We note that 16S microbiome analysis was found to be more sensitive for *Borrelia* detection than even nested PCR [23] so it is possible that some of the reads in the current study represent other *Borrelia* species that occur at very low relative abundance and may not be detectable by Sanger sequencing. Neither of the *Borrelia* sequences in this study was a close match to *B*. *lonestari* (~95.4% identity), suggesting that *B*. *lonestari* was not present in the ticks we sampled. Among the ticks in which at least 500 *Borrelia* sequences were detected, two were adult *I*. *scapularis*, four were *I*. *scapularis* nymphs and one was an *A*. *americanum* nymph. Several additional samples produced small number of reads for this OTU (N = 17, 1–18 reads per sample, median of 2 reads per sample). Given that *A*. *americanum* can acquire, but cannot transmit, *B*. *burgdorferi* through bloodfeeding, we decided to include in our characterization of *Borrelia* occurrence only samples for which several hundred reads was detected per sample. Specific components of the *I*. *scapularis* microbiome have been linked with inhibition and facilitation of *B*. *burgdorferi* transmission [10, 69] and members of the *B*. *burgdorferi* (sensu lato) complex in particular have been shown to affect vector survival and behavior [19]. Whether any of the bacteria we detected in our samples play similar roles is unknown, but we did detect several bacterial genera that were positively associated with *Borrelia* spp. in *I*. *scapularis* (Table 2). Such an association could be spurious, or could result from inoculation of *Borrelia* and another taxon from a common host source. Alternatively, the association could indicate an underlying mechanism of facilitated infection or persistence in the tick; gut bacterial communities in *I*. *scapularis* can affect the ability of *B*. *burgdorferi* to colonize the gut [10, 69]. Such indicator species analyses can identify potential interacting microbial genera for future transmission and co-infection studies or *in vitro* analyses of species interactions.

## Conclusions

As more data on vector microbiota are collected and published, patterns emerge and become reinforced, allowing for a clearer picture of the factors that may affect the tick microbiome, and, ultimately, consequences of microbiome variation. Whereas data on tick microbiota are accumulating rapidly, there are relatively few studies where stratified sampling designs are used to partition microbiome variation in natural populations. We showed that tick microbiota are largely species-specific and tend to be dominated by a handful of highly abundant lineages. We also suggest, based on our findings and previous results, that bloodfeeding may serve to ‘reset’ the microbiome and that exposure to the local environment (including subsequent hosts) allows for recolonization of certain microbial taxa. We also suggest that some of the maternal microbiome may be vertically transmitted to egg masses, at least in the case of *I*. *scapularis*. Descriptive and hypothesis-driven analyses of tick microbiomes are paving the way for additional experimental and functional studies wherein the roles of, and interactions among, specific bacterial genera and species can be studied in detail. Given the potential effects of microbes on tick host-seeking behavior, pathogen transmission, fitness, and survival, it is critical to advance our understanding of tick-microbiome relationships and interactions.

## Supporting information

S1 Table Sample information for all ticks included in this study. Data include Sample identifier, tick species, tick life stage, sampling location and coordinates, sampling date, number of sequence reads, OTU richness, and OTU diversity (Shannon’s Index).
(XLSX)

S1 Dataset Weighted unifrac plot representing all samples included in the study. Metadata associated with this plot can be found in the dataset DOI: 10.13140/RG.2.2.15215.38560. The three axes account for 65.03% of the variation in this dataset. Regarding stage designations, “E” represents eggs, “F” represents engorged female *I*. *scapularis* or unengorged female *A*. *americanum*, “L” represents larval mass, “M” represents unfed males, “N” represents nymphs, and “UF” represents unfed female *I*. *scapularis*. Site names and geographic coordinates can be found in S1 Table.
(ZIP)
